# Gynecologists May Underestimate the Amount of Blood Loss during Total Laparoscopic Hysterectomy

**DOI:** 10.1155/2018/3802532

**Published:** 2018-12-16

**Authors:** Masakazu Sato, Minako Koizumi, Kei Inaba, Yu Takahashi, Natsuki Nagashima, Hiroshi Ki, Nao Itaoka, Chiharu Ueshima, Maki Nakata, Yoko Hasumi

**Affiliations:** ^1^Department of Obstetrics and Gynecology, Mitsui Memorial Hospital, Chiyoda-ku, Tokyo, Japan; ^2^Department of Obstetrics and Gynecology, Graduate School of Medicine, The University of Tokyo, Bunkyo-ku, Tokyo, Japan

## Abstract

**Background:**

We considered the possibility of underestimation of the amount of bleeding during laparoscopic surgery, and we investigated comparing the amount of bleeding between laparoscopic surgery and open surgery by considering the concentration of hemoglobin before and after surgery as indicators.

**Methods:**

The following procedures were included: A, surgery for ovarian tumor; B, myomectomy; and C, hysterectomy either by laparoscopic surgery or open surgery. Patients who underwent the above procedures in between January 1, 2010, and December 31, 2017, were enrolled. We identified 1749 cases (A: 90, B: 105, and C: 325 of open surgery and A: 667, B: 437, and C: 125 of laparoscopic surgery). We considered the sum as an estimation of blood loss during surgery and the change in the value of hemoglobin in laboratory testing one day before and after surgery.

**Results:**

During laparoscopic surgery, the measurements of blood loss included the following: A: 59.8 ml; B: 168.6 ml; and C: 206.8 ml. During open surgery, measurements of blood loss included the following: A: 130.7 ml; B: 236.7 ml; and C; 280.9 ml. The reduction of hemoglobin after surgery compared with that before surgery was less in laparoscopic surgery than that in open surgery in A and B; however, this reduction was not significantly different in C.

**Conclusion:**

Our results suggest that the estimation of the bleeding in A and B was appropriate; however, the estimation might be underestimated in C during laparoscopic surgery.

## 1. Introduction

Laparoscopic surgery is now widely used in various medical fields, and there is no doubt that laparoscopic surgery is less invasive than open surgery [1]. For instance, after laparoscopic surgery, patients feel less pain from wounds [2–4], and they can start walking smoothly after surgery and discharge from the hospital earlier than those who underwent open surgery. The same is true for complications such as ileus. However, we raised the question as to the amount of blood loss that occurs during laparoscopic surgery. We agree that laparoscopic surgery needs delicate maneuvering, leading to less blood loss than open surgery [5–7]. However, we also considered the possibility of underestimation of the amount of bleeding. For instance, in the cases of gynecologic surgery, we could not always assess all the blood loss because of the Trendelenburg position, where the blood could rest in the abdominal cavity [8].

To address this issue, we investigated comparing the amount of bleeding between laparoscopic surgery and open surgery by considering the concentration of hemoglobin before and after surgery as indicators.

We investigated the representative operations in gynecological fields: A, surgery for ovarian tumor including cystectomy and salpingo-oophorectomy (unilateral or bilateral); B, myomectomy; and C, hysterectomy [9–11]. And our results suggest that the estimation of the bleeding in A and B was appropriate; however, it might be underestimated in C during laparoscopic surgery.

This study considered only the changes of the concentration of hemoglobin, and other ways of examining blood loss would be needed for further validation.

## 2. Materials and Methods

### 2.1. Patients

This study was approved by the Institutional Ethics Committee (Approval no. C77). Patients who underwent the following procedures between January 1, 2010, and December 31, 2017, were included in this study: A, surgery for ovarian tumor including cystectomy and salpingo-oophorectomy (unilateral or bilateral); B, myomectomy; and C, hysterectomy either by laparoscopy or open surgery. We sampled cases in which laboratory tests were obtained one day before and one day after surgery as part of a routine check-up. We excluded cases where measurements of blood loss could have been biased. In other words, the following cases were excluded: those requiring transfusion, with estimated blood loss exceeding 1000 ml; those who underwent lymphadenectomy; and those with peritoneal cancer and placement of drainage tubes. Overall, we identified 1749 cases: 520 cases (A: 90, B: 105, and C: 325) of open surgery and 1229 cases (A: 667, B: 437, and C: 125) of laparoscopic surgery. The cases wherein A was performed with B and C were assigned as B and C, respectively, resulting in no double counts. The median age and mean body weights of patients are as follows: A:37 years old (range, 15–85) and 54.7 ± 0.38 kg, B: 39 years old (range, 26–69) and 56.2 ± 0.61 kg, and C: 45 years old (range, 37–64) and 56.1 ± 1.1 kg in laparoscopic surgery and A:45 years old (range, 22–86) and 57.9 ± 1.48 kg, B: 41 years old (range, 29–50) and 57.9 ± 0.91 kg, and C: 48 years old (range, 36–84) and 57.6 ± 0.8 kg in open surgery.

### 2.2. Estimation of Bleeding and Change of the Concentration of Hemoglobin

We measured the grams of absorbed bleeding in gauze and suctioned bleeding, and we considered the sum as an estimation of blood loss during surgery [11, 12]. When we used saline for washing, we then subtracted the amount of saline from the estimation.

We routinely performed laboratory testing one day before and after surgery. For the change in the value of hemoglobin, we considered

(1) $$\begin{array}{c} \Delta \text{Hb}=\left(\frac{\left(\text{Hb\_pre–Hb\_post}\right)}{\text{Hb\_pre}}\right)*100\text{,} \end{array}$$

where Hb_pre is the concentration of hemoglobin one day before surgery and Hb_post is that one day after surgery. ΔHct (the changed value of hematocrit) was similarly defined.

### 2.3. Statistical Analysis

JMP 13 (SAS Institute, USA) was used for statistical analysis. Two-tailed *t*-test was used for comparing the means. *P* values less than 0.05 was considered as statistically significant. Values were described as means ± S.E.

## 3. Results

### 3.1. Measured Blood Loss and Change of the Concentration of Hemoglobin

**Figure 1:**
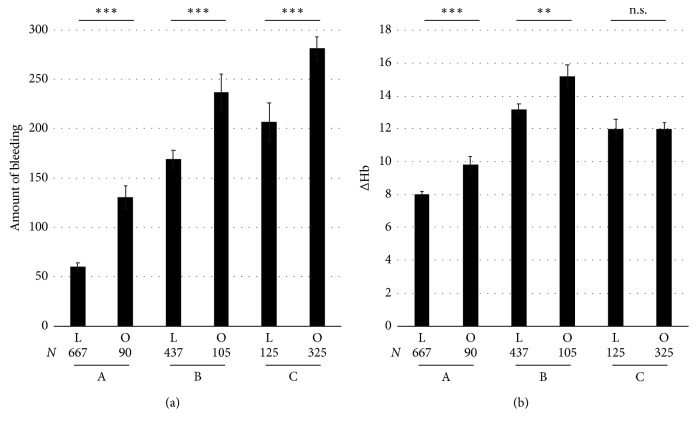
(a) Measurement of blood loss. The measurement of blood loss was A, 59.8 ± 4.2 ml; B, 168.6 ± 9.0 ml; and C, 206.8 ± 19.6 ml in laparoscopic surgery and A, 130.7 ± 11.7 ml; B, 236.7 ± 18.4 ml; and C, 280.9 ± 12.3 ml in open surgery. The blood loss was significantly lower in laparoscopic surgery than open surgery in A, B, and C (*p* < 0.0001, *p*=0.0009, and *p*=0.0014, respectively). (b) Change of the concentration of hemoglobin. The reduction of hemoglobin after surgery compared with that before surgery was A, 8.0 ± 0.2%; B, 13.3 ± 0.4%; and C, 12.0 ± 0.7% in laparoscopic surgery and A, 9.8 ± 0.6%; B, 15.2 ± 0.8%; and C, 12.0 ± 0.4% in open surgery. Blood loss was significantly lower during laparoscopic surgery than open surgery in A and B; however, it was not significantly different in C (*p*=0.0029, *p*=0.0243, and *p*=0.985, respectively). ΔHb=((Hb_pre − Hb_post)/(Hb_pre))*∗*100, where Hb_pre is the concentration of hemoglobin one day before surgery and Hb_post is that one day after surgery. ΔHct (the changed value of hematocrit) was similarly defined. A, surgery for ovarian tumor including cystectomy and salpingo-oophorectomy (unilateral or bilateral); B, myomectomy; C, hysterectomy; *N*, number of patients; L, laparoscopic surgery; O, open surgery. Values were described as means ± S.E. ^*∗*^ *p* < 0.05; ^*∗∗*^ *p* < 0.01; ^*∗∗∗*^ *p* < 0.001. n.s., not significant.

During laparoscopic surgery, the measurements of blood loss included the following: A: 59.8 ± 4.2 ml; B: 168.6 ± 9.0 ml; and C: 206.8 ± 19.6 ml. During open surgery, measurements of blood loss included the following: A: 130.7 ± 11.7 ml; B: 236.7 ± 18.4 ml; and C; 280.9 ± 12.3 ml (Figure 1(a)). The measured blood loss was significantly lower during laparoscopic surgery than during open surgery in A, B, and C (*p* < 0.0001, *p*=0.0009, and *p*=0.0014, respectively). During laparoscopic surgery, the reduction of hemoglobin after surgery compared with that before surgery was A: 8.0 ± 0.2%; B: 13.3 ± 0.4%; and C: 12.0 ± 0.7%. During open surgery, the reduction of hemoglobin was A: 9.8 ± 0.6%; B: 15.2 ± 0.8%; and C: 12.0 ± 0.4% (Figure 1(b)). This reduction of hemoglobin was significantly less during laparoscopic surgery than open surgery in A and B; however, it was not significantly different in C (*p*=0.0029, *p*=0.0243, and *p*=0.985, respectively). These results suggest that the estimation of bleeding in A and B was appropriate; however, the estimation might be underestimated in C. In other words, the measured blood loss in laparoscopic hysterectomy might be underestimated because the reduced value of hemoglobin after laparoscopic surgery was almost the same as that after open surgery.

## 4. Discussion

In the present study, we investigated the estimated amount of bleeding between laparoscopic surgery and open surgery, by calculating the changes of the concentration of hemoglobin before and after surgery as indicators.

**Figure 2:**
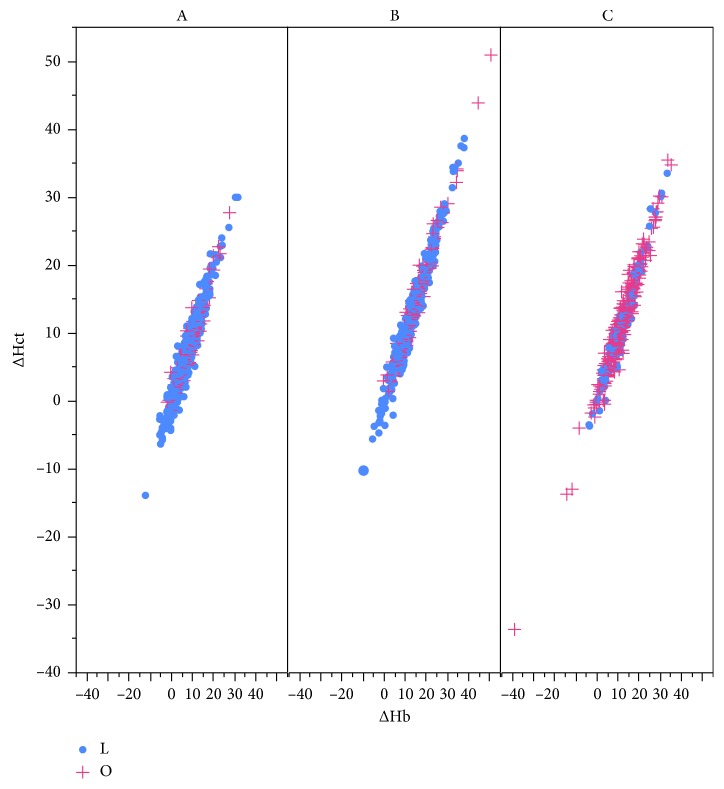
Correlation between the changes of hemoglobin and of hematocrit. The scatter plot patterns showed that the changed value of hemoglobin and hematocrit was not clearly different between laparoscopic surgery and open surgery. ΔHb=((Hb_pre − Hb_post)/(Hb_pre))*∗*100, where Hb_pre is the concentration of hemoglobin one day before surgery and Hb_post is that one day after surgery. ΔHct (the changed value of hematocrit) was similarly defined. A, surgery for ovarian tumor including cystectomy and salpingo-oophorectomy (unilateral or bilateral); B, myomectomy; C, hysterectomy; L, laparoscopic surgery; O, open surgery.

There is no doubt that laparoscopic surgery is less invasive than open surgery; however, we had the question about the accuracy of estimations of bleeding during laparoscopic surgery [1]. Of note, we could not always assess all of the blood loss due to the presence of Trendelenburg position in gynecologic surgery, where the bleeding could rest in the abdominal cavity [8]. To address this issue, we investigated comparing the amount of blood loss between laparoscopic surgery and open surgery by considering the concentration of hemoglobin before and after surgery as indicators. We investigated the representative operations in gynecological fields: A, surgery for ovarian tumors including cystectomy and salpingo-oophorectomy (unilateral or bilateral): B, myomectomy; and C, hysterectomy. We found that blood loss during laparoscopic hysterectomy might be underestimated because the reduction in the value of hemoglobin was almost the same as that in open surgery. The concentration of hemoglobin sometimes reduced noticeably due to massive intravenous drip infusions [13]. In these cases, the concentration of hematocrit could indicate dilution. However, as shown in Figure 2, the scatter plot patterns of the changed value of hemoglobin and hematocrit did not differ between laparoscopic surgery and open surgery. This finding means that compared with each other, it is probably less likely that differing approaches to surgery (laparoscopic surgery or open surgery) were influenced by the dilution or concentration of circulating blood.

This study was retrospective, and there was bias as to surgical approaches (choice of laparoscopic surgery or open surgery). In order to avoid the human error, we automatically abstracted the patient's information from the medical chart, and that is why we could only obtain simple back ground of the patients and could not consider the complication in detail in each patient. Generally, if we expected in advance difficulty in completing a laparoscopic surgery due to, for instance, adhesions, then we chose to perform open surgery. However, we think this bias may affect the bleeding during operation but may not affect the estimation itself. Indeed, we did not find any significant difference as to patients' body weight, which is known to affect the total blood volume (56.1 ± 1.1 kg in laparoscopic surgery and 57.6 ± 0.8 kg in open surgery) [11–13]. Thus, the fact that collected cases did not have significantly different changes in hemoglobin between laparoscopic surgery and open surgery but did have significantly different estimated amounts of blood loss still suggests the possibility that blood loss during laparoscopic hysterectomy might be underestimated.

How to estimate the bleeding during surgery is not different in essential between laparoscopic surgery and open surgery as described in Materials and Methods. And we think of two possibilities as to what could produce the discrepancy between laparoscopic hysterectomy and open hysterectomy. One is that we usually use a decent size of gauze and rubber spatula to keep the intestine in upper abdomen (out of the operational field). This might hold the blood in the operational field and help us to count the bleeding appropriately. The other is that we might not count all bleeding during laparoscopic surgery because aspirating the blood from the upper abdomen in Trendelenburg position is not always easy and sometimes skipped if the bleeding seemed not that much.

It might be a possible way to place drainage in all the patients and measure the amount of drained bleeding after surgery for the estimation of genuine blood loss, but this approach is still uncertain [14, 15]. Recently, some researchers have used computer imaging to detect the bleeding point [16]. The objective measuring way like that would be expected.

There is no doubt that laparoscopic surgery is less invasive than open surgery, but estimates of blood loss need to be carefully considered, at least in the case of laparoscopic hysterectomy. Our research may not directly affect the management in today's gynecologic surgery environment; however, it may be time to stop and think about minimally invasive surgery (MIS) because, for instance, MIS is now proposed to be associated with decreased survival compared to open radical hysterectomy among women with ≥2 cm stage IB1 cervical cancer [17]. Further research needs to be done to elucidate this topic.

## Data Availability

The raw data used to support the findings of this study are included within the supplementary information file.

## Supplementary Materials

Supplementary Materials Raw data including the amount of bleeding in each operation.

## References

[1] Dos Reis R., Andrade C., Frumovitz M., Munsell M., Ramirez P. T. (2018). Radical hysterectomy and age: outcomes comparison based on minimally invasive vs. Open approach. *Journal of Minimally Invasive Gynecology*.

[2] Aydogmus H., Aydoğmuş S., Gençdal S., Kelekçi S. (2017). Cuff closure by vaginal route in TLH: case series and review of literature. *Journal of Clinical and Diagnostic Research: JCDR*.

[3] Elessawy M., Schollmeyer T., Mettler L. (2014). The incidence of complications by hysterectomy for benign disease in correlation to an assumed preoperative score. *Archives of Gynecology and Obstetrics*.

[4] Istre O., Snejbjerg D. (2018). Complication rate of laparoscopic hysterectomies in Denmark, 2011–2016. *JSLS: Journal of Society of Laparoendoscopic Surgeons*.

[5] Bissolati M., Orsenigo E., Staudacher C. (2016). Minimally invasive approach to colorectal cancer: an evidence-based analysis. *Updates in Surgery*.

[6] Chue K. M., Goh G. H., Kow A. W. C. (2018). Right adrenal gland pseudocyst masquerading as a large symptomatic hepatic cyst: single incision laparoscopic (SILS) resection and a review of current literature. *Annals of Hepato-Biliary-Pancreatic Surgery*.

[7] Kasai M., Cipriani F., Gayet B. (2018). Laparoscopic versus open major hepatectomy: a systematic review and meta-analysis of individual patient data. *Surgery*.

[8] Ghomi A., Kramer C., Askari R., Chavan N. R., Einarsson J. I. (2012). Trendelenburg position in gynecologic robotic-assisted surgery. *Journal of Minimally Invasive Gynecology*.

[9] Alammari R., Lightfoot M., Hur H.-C. (2017). Impact of cystectomy on ovarian reserve: review of the literature. *Journal of Minimally Invasive Gynecology*.

[10] Beck T. L., Schiff M. A., Goff B. A., Urban R. R. (2018). Robotic, laparoscopic, or open hysterectomy - surgical outcomes by approach in endometrial cancer. *Journal of Minimally Invasive Gynecology*.

[11] Ju H., Hart R. A. (2016). Hidden blood loss in anterior lumbar interbody fusion (ALIF) surgery. *Orthopaedics and Traumatology: Surgery and Research*.

[12] Ram G. G., Suresh P., Vijayaraghavan P. V. (2014). Surgeons often underestimate the amount of blood loss in replacement surgeries. *Chinese Journal of Traumatology*.

[13] Gross J. B. (1983). Estimating allowable blood loss. *Anesthesiology*.

[14] Malik N. (2015). Review of one hundred consecutive abdominal hysterectomies: suitability for vaginal hysterectomy. *Journal of Ayub Medical College, Abbottabad: JAMC.*.

[15] Yavuzcan A., Basbug A., Bastan M., Caglar M., Ozdemir I. (2016). The effect of adenomyosis on the outcomes of laparoscopic hysterectomy. *Journal of Turkish German Gynecological Association*.

[16] Garcia-Martinez A., Vicente-Samper J. M., Sabater-Navarro J. M. (2017). Automatic detection of surgical haemorrhage using computer vision. *Artificial intelligence in medicine*.

[17] Jacob Margul D., Yang J., Seagle B. L., Kocherginsky M., Shahabi S. (2018). Outcomes and costs of open, robotic, and laparoscopic radical hysterectomy for stage IB1 cervical cancer. *Journal of Clinical Oncology*.

